# Modifying Role of GSTP1 Polymorphism on the Association between Tea Fluoride Exposure and the Brick-Tea Type Fluorosis

**DOI:** 10.1371/journal.pone.0128280

**Published:** 2015-06-05

**Authors:** Junhua Wu, Wei Wang, Yang Liu, Jing Sun, Yan Ye, Bingyun Li, Xiaona Liu, Hongxu Liu, Zhenqi Sun, Mang Li, Jing Cui, Dianjun Sun, Yanmei Yang, Yanhui Gao

**Affiliations:** Center for Endemic Disease Control, Chinese Center for Disease Control and Prevention, Harbin Medical University, Key Lab of Etiology and Epidemiology, Education Bureau of Heilongjiang Province & Ministry of Health (23618504), Harbin 150081, Heilongjiang Province, China; Children's National Medical Center, Washington, UNITED STATES

## Abstract

**Background:**

Brick tea type fluorosis is a public health concern in the north-west area of China. The association between SNPs of genes influencing bone mass and fluorosis has attracted attention, but the association of SNPs with the risk of brick-tea type of fluorosis has not been reported.

**Objective:**

To investigate the modifying roles of GSTP1 rs1695 polymorphisms on this association.

**Methods:**

A cross-sectional study was conducted. Brick-tea water was tested by the standard of GB1996-2005 (China). Urinary fluoride was tested by the standard of WS/T 89-2006 (China). Skeletal fluorosis was diagnosed by X-ray, the part we scheduled was forearm, shank, and pelvic, then diagnosed the skeletal fluorosis by the standard of WS/192-2008 (China). Gene polymorphism was tested by Sequenom MassARRAY system.

**Result:**

The prevalence rate in different ethnical participants was different: Tibetan individuals had the highest prevalence rate of skeletal fluorosis. There were significant differences in genotype frequencies of GSTP1 Rs1695 among different ethnical participants (p<0.001): Tibetan, Mongolian and Han subjects with homozygous wild type (GSTP1-AA) genotype were numerically higher than Kazakh and Russian subjects (*p*<0.001). Compared to Tibetan participants who carried homozygous A allele of GSTP1 Rs1695, Tibetan participants who carried G allele had a significantly decreased risk of skeletal fluorosis (OR = 0.558 [95% CI, 0.326-0.955]). For Kazakh participants, a decreased risk of skeletal fluorosis among carriers of the G allele was limited to non high-loaded fluoride status (OR = 0. 166 [95% CI, 0.035–0.780] vs. OR = 1.478 [95% CI, 0.866–2.552] in participants with high-loaded fluoride status). Neither SNP-IF nor SNP-age for GSTP1 Rs1695 was observed.

**Conclusion:**

The prevalence rate of the brick tea type fluorosis might have ethnic difference. For Tibetan individuals, who had the highest prevalence rate, G allele of GSTP1 Rs1695 might be a protective factor for brick tea type skeletal fluorosis.

## Introduction

Skeletal fluorosis is a chronic metabolic bone and joint disease, which is caused by long-term exposure to excessive amounts of fluoride [1]. It is a serious public health problem in many parts of the world where drinking water or foods contains more fluoride [1–5]. It is known that tea selectively absorbs and accumulates fluoride and high concentrations of fluoride have been reported in the tea drink of some areas [6–10]. Next to water, tea drink is the most commonly consumed beverage in the world [9, 11]. Black teas contain high levels of fluoride, and these concentrations of fluoride may represent a potential health hazard to tea drinkers [12–14]. The brick-tea type of fluorosis has been reported in minority regions of the western and northern parts of China, where brick-tea is a traditional drink and consumed in large quantities [15, 16]. An epidemiological survey, that was conducted in Naqu County, Tibet in September 2001 to investigate the manifestations of fluorosis in adults caused by the habitual consumption of brick tea, suggests that brick-tea type of fluorosis had even more severe adverse effects on human health compared with both the water-type and coal-burning type of fluorosis that occurred in other areas of China [17]. Moreover, the prevalence rate and severity of both the water-type and coal-burning type of fluorosis trended to decline, because of a series of preventive measures such as the defluoridation of drinking water, improvements in stoves, ventilation and grain-drying processes and so on in endemic fluorosis areas of China since the 1980s. However, brick-tea type of fluorosis is still a severe public health issue, because it is impossible to alter the brick-tea consumption among the minorities.

It is generally accepted that fluorosis is positively correlated to fluoride intake [18–20]. But not everyone with high fluoride exposure suffers fluorosis, what suggested that individual variation in fluorosis can exist when fluoride exposure is relatively constant in a community [21–22]. Fluorosis is characterized by clinical bone and tooth manifestations, resulting in skeletal fluorosis and dental fluorosis. Everett et al discovered that quantitative trait loci (QTL) on mouse chromosomes 2 and 11 influenced the variation in response to fluorosis [23–24]. Several studies reported that polymorphisms of COL1A2 [25], or estrogen receptor is related to the risk of dental fluorosis in children of high fluoride exposed population [26]. Recently, it was noticed that the myeloperoxidase gene polymorphism was related with fluorosis in adults living in the coal-burning endemic fluorosis area in Guizhou of China [27]. These results suggest that genetic factor may play an important role in the progress of fluorosis. However, the association of gene polymorphism with the risk of brick-tea type of fluorosis has not been reported.

Oxidative stress has been proposed to play an important role in the pathogenesis of the endemic fluorosis [28–29]. Glutathione S-transferases (GST) are phase II metabolizing enzymes that play an important role in cell protection by the clearance of harmful electrophilic compounds, including redox radicals. It was observed that the activity of GST pi class (GSTP1), encoded by GSTP1 gene, directly related with the clinical feature of fluorosis [30]. Functional significance has been demonstrated that differences in the expression and activity of GSTP1 have been related to single nucleotide polymorphisms including GSTP1 rs1695 (p.105 Ile > Val), resulting in reduced catalytic activity and detoxification capacity of the enzyme [2, 31–32].

It is clear that brick tea fluoride is the main source of adult fluorosis in minority regions of the western and northern parts of China, where people habitually drink black tea. However, no study has yet evaluated the modifying roles of GSTP1 rs1695 polymorphisms on this relationship. Therefore, we examined the modifying roles of GSTP1 rs1695 polymorphisms on this association.

## Materials and Methods

### Participants

A cross sectional study was conducted in sixteen villages of three provinces (Inner Mongolia, Qinghai, Sinkiang), People’s Republic of China, from July to August in 2012. Each of those villages was brick-tea type fluorosis village. The brick-tea type fluorosis village was defined as: people who over 16 years was greater than the average intake of tea fluoride 3.5mg/d, and had skeletal fluorosis confirmed by X-ray (GB17018, China). Villagers found being older than 16 years, birthed and grew up in the sixteen villages, were included in this cross sectional study. All of the participants filled in demography survey questionnaire, and received clinical examination which included physical examination and past medical history and X-ray diagnosis (Beijing Longsafe Imaging Technology Co., Beijing City, China). In addition, brick tea water, blood and urine was collected from each participant.

### Questionnaire

The questionnaire was designed to obtain demographic information, including name, address, sex, age, personal or family history of bone related disease, education, economic income, type of brick tea and the amount of drinking brick tea water per day and drinking duration. The investigation was performed face to face by well-trained staff.

### Diagnose of skeletal fluorosis

The skeletal fluorosis was diagnosed by the Chinese Diagnostic Criteria of Endemic skeletal fluorosis (WS192-2008, China). An individual’s skeletal fluorosis was based on the sign of X-ray, including osteoporosis, osteomalacia, sclerosis, turnover, ossification of soft tissue and joint degeneration in the forearm, shank, and pelvic, and could be classified into three parts: mild, moderate and severe. **Mild**: i) The normal trabecular pattern was replaced with gravel-like or granular bone spot; or ii) The bone trabecula became thinner and sparse, and the spatial arrangement was irregular and obscuring; or iii) The hardened zone presented in the metaphysis of the long bone and the slight ossification presented in the soft tissue around forearm or lower leg bone; or iv) The Radial crest was enlarged and the border was hardened；v)The interosseous membrane in the forearm or lower leg bone was ossificated, which showed the sign of bud break ground. **Moderate**: i) The bone trabecula presented extensive diffused coarse dense fusion; or ii) Osteoporosis presented extensive, and the interosseous membrane in the forearm or lower leg bone was ossificated; or iii) The trabecular structure in the metaphseal of limbs was obviously disorder and obscuring, and osteoporosis presented in the cortical bone of teretipronator attachment.iv) Obvious ossification presented in the tendon and ligament of the pelvis and the interosseous membrane of the forearm or lower leg bone. **Severe**: i) Most bone trabecula fused and presented dentin-like osteosclerosis; or ii) Osteoporosis or osteomalacia presented obviously, and the interosseous membrane in the forearm or lower leg bone was ossificated; or iii) Bone trabecula presented broken felt-like, bone structure presented cotton-like, cortical bone was ossificated, bone density increased and ostealleosis presented; or iv) Multiple large joints presented severe degenerative changes and deformity, and the soft tissue around the bone obviously ossificated.

### Fluoride analysis

The brick tea water sample or urine sample was stored at -20°C until use. The concentration of tea water fluoride was detected by F-ion selective electrode (Yingke Crystal Materials Company) with a national standardized method in China (GB19965-2005, China). All the samples were analyzed twice, and the means of two results were standing for the finally fluoride concentration. The test method of urinary fluoride was as the standard of WS/T 89–2006 (China). According to standard of WS/T 256–2005 (China), the participants with a fluoride concentration exceeding 1.6 mg⁄ L in the urine were classified as highly exposed.

### GSTP1 genotyping

Genomic DNA was extracted from the blood samples with DNA extraction kit (Axygen Biosciences, Union City, USA), we tested the DNA concentration by TU1901 Spectrophotometry (Purkinje General Company, Beijing City, China) to make sure the DNA concentration was greater than 20 μg/ml. The extracted genomic DNA was stored at -80°C until use. All of the genotyping sequencing from the extract were performed by the Shanghai Fenglin Clinical Laboratory Company (http://www.fenglinlab.com/index.asp) using the Sequenom MassARRAY system (Sequenom, Inc., San Diego, CA, USA). The primer sequence of GSTP1 Rs1695: forward -5′- ACGTTGGATGTGGTGGACATGGTGAATGAC-3′, reverse-5′-ACGTTGGATGTGGTGCAGATGCTCACATAG-3′, extended-5′- GTTGGTGTAGATGAGGGAGA-3′. For the genotyping sequencing quality control, blinded blood duplicate was used.

### Statistical analysis

The database was shown in S1 Dataset. All statistical analyses were performed with the SPSS version 18.0 (SPSS Inc., Chicago, IL). Pearson’s chi-square test was used for test of the differences between fluorosis patients and control people. Odds ratios (OR) and corresponding 95% confidence intervals (CI) were calculated for skeletal fluorosis risk using logistic regression. Testing for deviation from Hardy-Weinberg equilibrium (HWE) was performed stratified by ethnicity using a chi-square test. Gene-environment interactions were conducted using Wald’s test statistic. *P*<0.05 was considered statistically significant.

### Ethical statement

The study was approved by the Ethical Review Board of Harbin Medical University (HMUIRB20120021). All of those participants signed informed consent, and we also obtained written informed consent from the guardians on behalf of the minors. No specific permits were required for the locations or activities associated with the brick-tea water sample collection in this field study. The locations were not privately owned or protected in any way and this field study did not involve endangered or protected species.

## Result

### Participant characteristics

**Fig 1:**
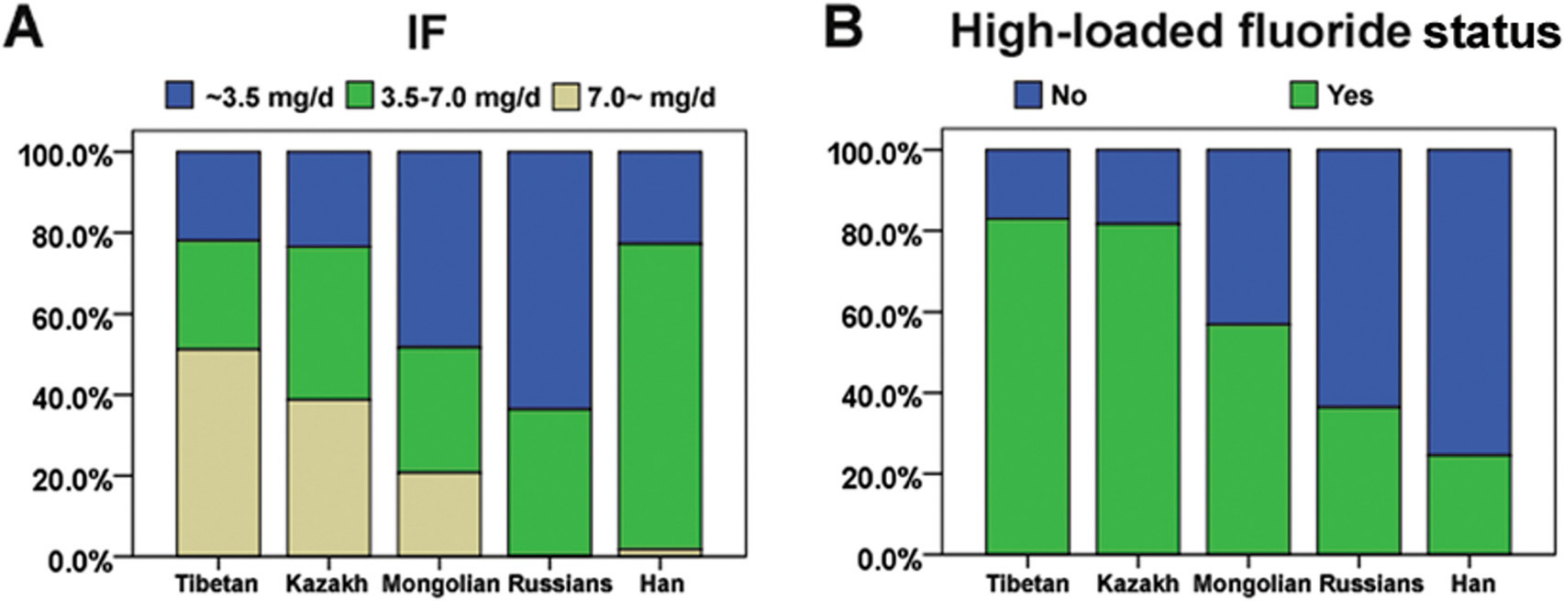
The fluoride exposure and IF in different ethnical brick tea type of fluorosis.

Descriptive statistics of skeletal fluorosis cases and controls were presented overall and stratified by ethnicity in Table 1. Skeletal fluorosis cases were significantly older than control (p<0.001); this difference was observed separately in Tibetan (p<0.001), Kazakh participants (p = 0.0143), Mongolian participants (p = 0.014) and Han participants (p = 0.002). There were more male in the skeletal fluorosis cases than in the controls (p = 0.022); this difference was only observed in Tibetan participants (p = 0.018). Skeletal fluorosis cases were more likely to have increased fluoride exposure (p<0.001), this difference was separately observed in Tibetan participants (p = 0.004) and Mongolian participants (p = 0.018). Skeletal fluorosis cases were more likely to have a more IF (p<0.001), this difference was separately observed in Tibetan participants (p = 0.011), Mongolian participants (p = 0.015) and Han participants (p = 0.03). In addition, significant differences were observed between different ethnical cases, such that a greater percentage of Tibetan cases and Kazakh cases had more fluoride exposure, had an IF more than 7.0 mg/L (Fig 1).

**Table 1:** Descriptive characteristics of study participants (N = 1414).

	Cases^a^						Controls^a^							
	All n (%) n = 347	Tibetan n (%) n = 123	Kazakh n (%) n = 98	Mongolian n (%) n = 58	Han n (%) n = 57	Russians n (%) n = 11	All n (%) n = 1067	Tibetan n (%) n = 185	Kazakh n (%) n = 192	Mongolian n (%) n = 203	Han n (%) n = 368	Russians n (%) n = 119	*p* ^*^	*p* ^#^
**Age**													<0.001	0.033
~45	60 (17.3)	20 (16.3)	20 (20.4)	14 (24.1)	5 (8.8)	1 (9.1)	372 (34.9)	78 (42.2)	66 (34.4)	91 (44.8)	96 (26.1)	41 (34.5)		
46~65	167 (48.1)	45 (36.6)	51 (52.0)	37 (63.8)	27 (47.4)	7 (63.6)	476 (44.6)	72 (38.9)	82 (42.7)	92 (45.3)	172 (46.7)	58 (48.7)		
66~	120 (34.6)	58 (47.2)	27 (27.6)	7 (27.3)	25 (43.9)	3 (27.3)	219 (20.5)	35 (18.9)	44 (22.9)	20 (9.9)	100 (27.2)	20 (16.8)		
**Sex**													0.022	0.499
Male	161 (46.4)	64 (52.0)	44 (44.9)	22 (37.9)	26 (45.4)	5 (45.5)	421 (39.5)	71 (38.4)	65 (33.9)	81 (39.9)	151 (41.0)	53 (44.5)		
Female	186 (53.6)	59 (48.0)	54 (55.1)	36 (62.1)	31 (54.4)	6 (54.5)	646 (60.5)	114 (61.6)	127 (66.1)	122 (60.1)	217 (59.0)	66 (55.5)		
**Ethnicity**													<0.001	-
Tibetan	123 (35.4)	-	-	-	-	-	185 (17.3)	-	-	-	-	-		
Kazakh	98 (28.2)	-	-	-	-	-	192 (18.0)	-	-	-	-	-		
Mongolian	58 (16.7)	-	-	-	-	-	203 (19.0)	-	-	-	-	-		
Han	57 (16.4)	-	-	-	-	-	368 (34.5)	-	-	-	-	-		
Russians	11 (3.2)	-	-	-	-	-	119 (11.2)	-	-	-	-	-		
**IF**													<0.001	<0.001
~3.5 mg/d	98 (28.2)	27 (22.0)	23 (23.5)	28 (48.3)	13 (22.8)	7 (63.6)	367 (34.4)	41 (22.2)	45 (23.4)	82 (40.4)	141 (38.3)	58 (48.7)		
3.5–7.0 mg/d	135 (38.9)	33 (26.8)	37 (37.8)	18 (31.0)	43 (75.4)	4 (36.4)	503 (47.1)	78 (42.2)	57 (29.7)	101 (49.8)	210 (57.1)	57 (47.9)		
7.0~ mg/d	114 (32.9)	63 (51.2)	38 (38.8)	12 (20.7)	1 (1.8)	0 (0)	197 (18.5)	66 (35.7)	90 (46.9)	20 (9.9)	17 (4.6)	7 (4)		
**High-loaded fluoride status**													<0.001	<0.001
Yes	233 (67.1)	102 (82.9)	80 (81.6)	33 (56.9)	14 (24.6)	4 (63.6)	488 (45.7)	126 (68.1)	168 (87.5)	80 (39.4)	80 (21.7)	34 (28.6)		
No	114 (32.9)	21 (17.1)	18 (18.4)	25 (43.1)	43 (75.4)	7 (36.4)	579 (54.3)	59 (31.9)	24 (12.5)	123 (60.6)	288 (78.3)	85 (71.4)		
**Skeletal fluorosis grade**														<0.001
mild	259 (74.6)	68 (55.3)	82 (83.7)	47 (81.0)	52 (91.2)	10 (90.9)	-	-	-	-	-		-	
moderate	59 (17.0)	32 (26.0)	11 (11.2)	10 (11.2)	5 (8.8)	1 (9.1)	-	-	-	-	-			
severe	29 (8.4)	23 (18.7)	1 (5.1)	5 (5.1)	0 (0)	0 (0)	-	-	-	-	-			

a Percentages are adjusted for sampling weights and may not sum to 1 due to rounding; p*, p value difference by case status; *p* ^#^, p value difference by ethnicity (in case)

There were 347 subjects who were diagnosed with skeletal fluorosis. The prevalence rate in different ethnical participants was listed in Table 2. Tibetan participants had the highest prevalence rate of skeletal fluorosis even after adjustment for known risk factors (age or sex).

**Table 2:** The prevalence rate in different ethnical participants.

	Tibetan	Kazakh	Mongolian	Han	Russians
Crude prevalence rate	39.9%	33.8%	22.2%	13.4%	8.5%
Prevalence rate after adjustment for age	38.7%	33.7%	23.3%	12.5%	8.8%
Prevalence rate after adjustment for sex	39.6%	34.2%	22.2%	13.4%	8.5%
Prevalence rate after adjustment for age and sex	39.3%	33.2%	23.8%	12.7%	8.4%

### Allele and genotype frequencies of GSTP1 Rs1695

Table 3 showed the allele and genotype frequencies of GSTP1 Rs1695 in different ethnical participants. All ethnical participants were found to be in Hardy-Weinberg equilibrium for GSTP1 Rs1695. There were significant differences in genotype frequencies of GSTP1 Rs1695 among different ethnical participants (p<0.001). Tibetan, Mongolian and Han subjects with homozygous wild type (GSTP1-AA) genotype were numerically higher than Kazakh and Russian subjects (*p*<0.001).

**Table 3:** Genotype and allele frequencies of GSTP1 Rs1695 by ethnicity.

		Genotype					
	n	AA	AG	GG	p*	MAF (%)	HWE *P*
Tibetan	308	208	95	5	0.001	17.0	0.112
Kazakh	290	158	113	19		26.0	0.841
Mongolian	261	173	79	9		18.6	0.996
Han	425	294	118	13		16.9	0.782
Russians	130	74	52	4		23.1	0.149

HWE, Hardy Weinberg Equilibrium; MAF, minor allele frequency; p*, p value difference by ethnicity

### Association of GSTP1 Rs1695 polymorphisms with skeletal fluorosis

Because of low frequency of G alleles, we divided the subjects into two groups by the presence and absence of G allele, that is, AG/GG and AA groups. Compared to Tibetan participants who carried homozygous A allele of GSTP1 Rs1695, Tibetan participants who carried G allele had a significantly decreased risk of skeletal fluorosis, however, similar reduction in risk did not find shared by other ethnical participants (Table 4).

**Table 4:** Risk of skeletal fluorosis associated with GSTP1 Rs1695 in subjects overall and stratified by ethnicity.

	Case (n)	Control (n)	crude OR (95% CI)	adjusted OR (95% CI)*
**All subjects**				
AA	226	681	1.0 (ref)	1.0 (ref)
AG+GG	121	386	0.945 (0.733, 1.217)	0.907 (0.693,1.066)
**Tibetan**				
AA	91	117	1.0 (ref)	1.0 (ref)
AG+GG	32	68	**0.605 (0.366, 0.999)**	**0.558 (0.326,0.955)**
**Kazakh**				
AA	52	106	1.0 (ref)	1.0 (ref)
AG+GG	46	86	1.090 (0.669, 1.776)	1.082 (0.659,1.776)
**Mongolian**				
AA	36	137	1.0 (ref)	1.0 (ref)
AG+GG	22	66	1.269 (0.692, 2.326)	1.225 (0.657,2.283)
**Han**				
AA	39	255	1.0 (ref)	1.0 (ref)
AG+GG	18	113	1.042 (0.571, 1.899)	1.116 (0.605,2.057)
**Russians**				
AA	8	66	1.0 (ref)	1.0 (ref)
AG+GG	3	53	0.467 (0.118,1.847)	0.446 (0.108,1.843)

* Adjusted for age, sex, ethnicity, and fluoride exposure.

We investigated the potential for GSTP1 Rs1695 SNP-fluoride exposure interactions (Table 5). This interaction was only apparent in for Kazakh participants (*p* = 0.004) but not other ethnical participants (*p*>0.05). For Kazakh participants, a decreased risk of skeletal fluorosis among carriers of the G allele was limited to non high-loaded fluoride status (OR = 0. 166 [95% CI, 0.035–0.780] vs. OR = 1.478 [95% CI, 0.866–2.552] in participants with high-loaded fluoride status).

**Table 5:** Risk of skeletal fluorosis associated with GSTP1 Rs1695 in subjects, stratified by fluoride exposure levels.

	Non high-loaded fluoride status			High-loaded fluoride status			
	Case (n)	Control (n)	OR (95% CI)*	Case (n)	Control (n)	OR (95% CI)*	*p*
**All subjects**							0.204
AA	82	376	1.0 (ref)	144	305	1.0 (ref)	
AG+GG	32	203	0.736 (0.463, 1.168)	89	183	1.031 (0.737, 1.442)	
**Tibetan**							0.445
AA	16	34	1.0 (ref)	75	83	1.0 (ref)	
AG+GG	5	25	0.426 (0.129, 1.406)	27	43	0.622 (0.339, 1.144)	
**Kazakh**							**0.004**
AA	15	11	1.0 (ref)	37	95	1.0 (ref)	
AG+GG	3	13	**0.166 (0.035, 0.780)**	43	74	1.487 (0.866, 2.552)	
**Mongolian**							0.789
AA	17	86	1.0 (ref)	18	51	1.0 (ref)	
AG+GG	8	37	1.043 (0.408, 2.669)	14	29	1.359 (0.581, 3.183)	
**Han**							0.602
AA	30	198	1.0 (ref)	9	57	1.0 (ref)	
AG+GG	13	90	1.140 (0.554, 2.343)	5	23	1.536 (0.448, 5.268)	
**Russians**							0.139
AA	4	47	1.0 (ref)	4	19	1.0 (ref)	
AG+GG	3	38	1.260 (0.243, 6.550)	0	15	-	

* Adjusted for age, sex, ethnicity, and IF. p value testing heterogeneity.

We also investigated the potential for SNP-IF and SNP-age for GSTP1 Rs1695. Although a decreased risk of skeletal fluorosis in Kazakh carriers of the G allele was limited to Tibetan participants aged 45 to 65 years (OR = 0. 374 [95% CI, 0.180–0.777]), the test of heterogeneity was not statistically significant (p = 0.160, Table 6). This interaction of GSTP1 Rs1695 with fluoride exposure status did not be observed (Table 7).

**Table 6:** Risk of skeletal fluorosis associated with GSTP1 Rs1695 in subjects, stratified by age.

	~45			46~65			66~			
	Case(n)	Control(n)	OR (95% CI)*	Case(n)	Control(n)	OR (95% CI)*	Case(n)	Control(n)	OR (95% CI)*	*p*
**All subjects**										0.292
AA	35	244	1.0 (ref)	136	352	1.0 (ref)	55	85	1.0 (ref)	
AG+GG	25	128	1.410 (0.793, 2.507)	74	211	0.844 (0.596, 1.193)	22	47	0.671 (0.346, 1.301)	
**Tibetan**										0.160
AA	15	53	1.0 (ref)	51	49	1.0 (ref)	25	15	1.0 (ref)	
AG+GG	5	25	0.640 (0.200, 2.046)	15	38	**0.374 (0.180, 0.777)**	12	5	1.537 (0.427, 5.529)	
**Kazakh**										0.531
AA	10	39	1.0 (ref)	33	53	1.0 (ref)	9	14	1.0 (ref)	
AG+GG	10	27	1.593 (0.569, 4.458)	30	43	1.107 (0.583, 2.103)	6	16	0.874 (0.215, 3.549)	
**Mongolian**										0.119
AA	6	65	1.0 (ref)	36	63	1.0 (ref)	5	9	1.0 (ref)	
AG+GG	8	26	2.980 (0.855, 10.383)	13	25	0.981 (0.437, 2.204)	1	4	0.281(0.020, 3.953)	
**Han**										0.176
AA	4	64	1.0 (ref)	21	151	1.0 (ref)	14	40	1.0 (ref)	
AG+GG	1	32	0.791 (0.072, 8.740)	15	66	1.467 (0.702, 3.066)	2	5	0.342(0.066, 1.771)	
**Russians**										0.208
AA	0	23	1.0 (ref)	6	36	1.0 (ref)	2	7	1.0 (ref)	
AG+GG	1	18	-	1	28	0.236 (0.025, 2.212)	1	7	0.395 (0.020, 7.866)	

Adjusted for sex, ethnicity, IF and fluoride exposure. p value testing heterogeneity.

**Table 7:** Risk of skeletal fluorosis associated with GSTP1 Rs1695 in subjects, stratified by IF levels.

	~3.5mg/L			3.5mg/L~7.0mg/L			7.0mg/L~			
	Case(n)	Control(n)	OR (95% CI)*	Case(n)	Control(n)	OR (95% CI)*	Case(n)	Control(n)	OR (95% CI)*	*p*
**All subjects**										0.404
AA	63	251	1.0 (ref)	90	314	1.0 (ref)	73	116	1.0 (ref)	
AG+GG	35	116	1.202 (0.733, 1.973)	45	189	0.833 (0.544, 1.276)	41	81	0.827 (0.498, 1.375)	
**Tibetan**										0.252
AA	23	27	1.0 (ref)	24	44	1.0 (ref)	44	46	1.0 (ref)	
AG+GG	4	14	0.289 (0.078, 1.068)	9	34	0.575 (0.216, 1.528)	10	20	0.826 (0.363, 1.881)	
**Kazakh**										0.779
AA	12	28	1.0 (ref)	21	34	1.0 (ref)	19	44	1.0 (ref)	
AG+GG	11	17	1.551 (0.548, 4.383)	16	23	0.937 (0.377, 2.332)	19	46		
**Mongolian**										0.283
AA	15	55	1.0 (ref)	11	69	1.0 (ref)	10	13	1.0 (ref)	
AG+GG	13	27	1.673 (0.680, 4.114)	7	32	1.443 (0.487, 4.271)	2	7	0.402 (0.056, 2.869)	
**Han**										0.262
AA	9	106	1.0 (ref)	30	137	1.0 (ref)	0	12	1.0 (ref)	
AG+GG	4	35	1.058 (0.272, 4.121)	13	73	0.903 (0.437, 1.866)	1	5	_	
**Russians**										0.093
AA	4	35	1.0 (ref)	4	30	1.0 (ref)	0	1	1.0 (ref)	
AG+GG	3	23	1.082 (0.209, 5.592)	0	27	—	0	3	_	

Adjusted for age, sex, ethnicity, and fluoride exposure. p value testing heterogeneity.

## Discussion

Brick tea type fluorosis is a public health concern in the north-west area of China due to the excessive consumption of fluoride in brick-tea infusion [14–15]. As the similarities in size and charge, fluoride replaces the hydroxyl ion in the crystal lattice of apatite, resulting that approximately 98% of the fluoride in the body is associated with calcified tissues [33]. Fluoroapatite is less soluble, more compact, and slower to undergo remodeling in bone [34], so excessive intake of fluoride may affect both bone metabolism and enamel development, causing skeletal and dental fluorosis, respectively. Although it is generally accepted that high fluoride intake is the main risk factor for fluorosis, there have been several indications of a potential influence of genetic factors on susceptibility to fluorosis. Murine studies established that 12 inbred strains of mice showed different susceptibilities to dental fluorosis: the A/J mouse strain is highly susceptible, with a rapid onset and severe development of dental fluorosis, whereas the 129P3/J mouse strain is least affected, with minimal dental fluorosis [35–36]. Epidemiological studies have found racial/ethnic differences in fluorosis prevalence that could not be explained by levels of fluoride intake [37]. In our study, we observed that the prevalence rate in different ethnical participants was different: Tibetan participants had the highest prevalence rate of skeletal fluorosis even after adjustment for known risk factors, but the fluoride exposure and IF is similar between Tibetan and Kazakh participants. These observations indirect support the possible contribution of a genetic component in the pathogenesis of fluorosis.

Single nucleotide polymorphism (SNP), a kind of DNA polymorphism in genome which results from the variation of single nucleotide, underlies the differences in susceptibility to disease. In 2009, Everett et al detected 354 SNPs distributed throughout the genome of the young mice inherited from the fluoride susceptible and fluoride resistant parents and discovered a significant evidence indicating that the QTL on chromosomes 2 and 11 influenced the variation in response to dental fluorosis [23]. Afterwards, the association between SNPs of genes influencing bone mass and fluorosis has attracted attention [25–27, 38–39]. Most studies focus on the contribution of SNPs on the dental fluorosis, but the association of SNPs with the risk of brick-tea type of fluorosis has not been reported. Excessive fluoride exposure causes oxidative stress, which has been observed in population living in endemic fluorosis areas [28–29]. The activity of GSTP1, a phase II metabolizing enzyme, directly related with the clinical feature of fluorosis [30]. Chromosome QTL 11q12-13, where the GSTP1 gene is located, has been associated with variation of bone mineral density (BMD) [40–41] and osteoporosis-pseudoglioma syndrome, a disorder affecting skeletal strength and vision [42]. The GSTP1 rs1695 SNP, which located within the active site of the enzyme, alters the substrate specificity, activity and thermostability of the GSTP enzyme [2, 31–32]. It was reported that the genotype and allele frequencies of GSTP1 rs1695 are different in different ethnic [43]. In this study, we observed that there were significant differences in genotype frequencies of GSTP1 Rs1695 among different ethnical participants: the G allele was least common among Tibetan individuals. As mentioned above, Tibetan participants had the highest prevalence rate of skeletal fluorosis. Therefore, we investigated the association between GSTP1 Rs1695 SNP and the susceptibility to brick-tea type of fluorosis.

In the present study, we observed no relationship between GSTP1 Rs1695 SNP and brick-tea type of fluorosis overall, but Tibetan individuals with G allele of GSTP1 Rs1695 had a significantly decreased risk of skeletal fluorosis compared to Tibetan individuals with homozygous A allele after adjusting for age, sex, IF and fluoride exposure status. Evidence has demonstrated that individuals with G allele of GSTP1 Rs1695 have lower thermal stability and lower activities to a variety of substrates compared individuals with homozygous A allele [43–44]. Therefore, the reduced risks of brick-tea type of fluorosis associated with G allele of GSTP1 Rs1695 are consistent with the evidence indicating that the activity of GSTP1 directly related with the clinical feature of fluorosis [30]. However, the patient from other origins carried the same allele did not present resistance by the statistical analysis. It seemed that the gene polymorphism of GSTP1 Rs1696 was a necessary but not sufficient condition for skeletal fluorosis, which might be interfered by other factors, such as fluoride exposure.

It is known that age, sex, and dose and duration of fluoride intake are the major factors that influence the fluoride toxicity in humans and bring about variations in the clinical presentation [1]. So, the potential for GSTP1 Rs1695 SNP-known risk factor interactions were further investigated overall or by ethnic. We only observed that a decreased risk of skeletal fluorosis among carriers of the G allele was limited to Kazakh individuals with non high-loaded fluoride status, suggesting that high-loaded fluoride would compromise the protective effect of the G allele of GSTP1 Rs1695. However, the findings of the SNP analysis stratified by fluoride exposure status need to be interpreted with caution because the data became sparse when several factors were investigated simultaneously.

## Conclusions

In summary, in this study, we find the ethnic difference in prevalence rate of the brick tea type fluorosis and Tibetan individuals had the highest prevalence rate. For Tibetan individuals, G allele of GSTP1 Rs1695 might be a protective factor for brick tea type skeletal fluorosis. However, fluorosis is a complex disease and it is likely that several genes and/or polymorphic sites influence its malformations. Moreover, our sample size of each race was too small to had high precision. Therefore, further investigation on other polymorphisms of candidate genes influencing bone mass may be useful.

## Supporting Information

S1 Dataset Data of this study. (XLS)
